# Cognitive behaviour therapy-based early intervention and prevention programme for anxiety in South African children with visual impairments

**DOI:** 10.4102/ajod.v10i0.796

**Published:** 2021-01-29

**Authors:** Lisa Visagie, Helene Loxton, Leslie Swartz, Paul Stallard

**Affiliations:** 1 Department of Psychology, Faculty of Arts and Social Sciences, Stellenbosch University, Stellenbosch, South Africa; 2 Department for Health, Faculty of Humanities and Social Sciences, University of Bath, Claverton Down, Bath, United Kingdom

**Keywords:** anxiety, prevention, cognitive behaviour therapy, visual impairment, South Africa, children, schools-based interventions, efficacy

## Abstract

**Background:**

Anxiety is the most common psychological difficulty reported by youth worldwide and may also be a significant problem for children with visual impairments. Cognitive behaviour therapy (CBT) interventions have proven to be successful in treating childhood anxiety; however, mostly these are not suitable for children with visual impairments, as the materials used are not sufficiently accessible to this population.

**Objectives:**

The present study was motivated by the dearth of research on this topic and aimed to examine the effects of a specifically tailored, group-based, universally delivered, CBT intervention for anxiety in children with visual impairments and to examine the influence of three predictor variables (i.e. age, gender and level of visual impairment) on prevention effects.

**Method:**

A randomised wait-list control group design with pre-, post- and follow-up intervention measures was employed. The final sample of 52 children (aged 9–14) with varying degrees of visual impairment received the anxiety intervention. Participants were followed over a course of 10 months during which their anxiety symptoms were assessed quantitatively at four time points (T1–T4).

**Results:**

The results indicated that the anxiety intervention did not significantly decrease symptoms of anxiety within the intervention groups. However, the intervention appeared beneficial for girls, younger children and legally blind participants.

**Conclusion:**

This study demonstrated how CBT interventions can be adapted for use in children with visual impairments. Results obtained provide a foundation upon which future updated anxiety intervention programmes can be built, meeting the need for further research in this area.

## Introduction

Childhood anxiety is common, with most anxiety being functional and beneficial, providing motivation and preventing excessive risk-taking. However, for a number of children, anxiety has a negative effect and interferes so significantly in their daily functioning, or is so developmentally inappropriate, that the diagnosis of an anxiety disorder may be warranted (Campbell 2003). If left untreated, anxiety symptoms persist and increase in severity (Prinzie et al. 2014; Weems & Silverman 2013) placing children at risk for depression, substance abuse, higher suicide ideation, illicit drug dependence, higher rates of school drop-out and unemployment in late adolescence and adulthood (Ahlen et al. 2012; Bittner et al. 2007; Donovan & Spence 2000; McLoone, Hudson & Rapee 2006; Stallard 2010; Stallard et al. 2007).

Children with physical disabilities are more prone than their non-disabled peers to the development of psychological difficulties (including anxiety) (Gullone 1996; Ollendick, Matson & Helsel 1985), and a particular high-risk group are children with visual impairments (Visagie et al. 2013). Despite this increased risk, children with visual impairments have been neglected in previous fear and anxiety research. The last international study on this research topic was conducted by Weimer and Kratochwill (1991) in America more than two decades ago. Given their increased ‘risk’, it is important to develop appropriate interventions to equip visually impaired children with the necessary skills and strategies to manage anxiety symptoms when they arise.

Cognitive behaviour therapy (CBT) is a well-established, highly researched, evidence-based intervention for the treatment and prevention of anxiety (Silverman, Pina & Viswesvaran 2008; Walkup et al. 2008). However, despite CBT’s encouraging outcomes, less than one quarter of children who experience anxiety difficulties receive treatment (Korkodilos 2016; Lawrence et al. 2015; Merikangas et al. 2010), and of those who do, many will terminate treatment prematurely (Pina et al. 2003; Wergeland et al. 2015), fail to respond (Rey, Marin & Silverman 2011) or continue to experience recurrent difficulties despite treatment (Last et al. 1996). A potential strategy to overcome these limitations, and manage the high prevalence of anxiety and its negative consequences, is to place greater focus on anxiety prevention (Johnston, Kemps & Chen 2018). Prevention programmes aim to reduce the incidence or onset of mental health disorders by reducing risk factors and developing protective factors to prevent the development of these disorders (World Health Organization 2004). Prevention programmes are usually conceptualised by their intended focus, either indicated (targeted to participants displaying sub-clinical or mild symptoms of disorder), selective (targeted to participants identified as being at risk of developing a particular disorder) or universal (targeted towards whole populations regardless of risk status) (Liddle & Macmillan 2010; Mrazek & Haggerty 1994; World Health Organization 2004). Universally delivered CBT-based anxiety prevention programmes have shown very positive results; however, they have focused primarily on children without disabilities (see Johnston et al. 2018 for an overview). The most well-known CBT group-based anxiety prevention programmes are Kendall’s (1990) Coping Cat programme and Barrett’s (2005) FRIENDS programme. Although CBT-based anxiety intervention programmes have reported promising outcomes, these programmes are not suitable for children with visual impairments, as much of their content relies on visual presentation or representations (i.e. printed worksheets, cartoons, pictures and video-material). To the researchers’ knowledge, there is currently no anxiety intervention programme tailored to meet the specific needs of children with visual impairments. Considering the given factors and the notion that children with visual impairments are possibly more prone to the development of anxiety (Visagie et al. 2013), the researchers and colleagues (Visagie 2016; Visagie, Loxton & Silverman 2015; Visagie et al. 2017) developed and implemented a specifically tailored CBT-based anxiety intervention programme (Positive and Motivating programme – PAM) for South African visually impaired children.

This article aims to overcome gaps in the childhood anxiety literature and presents preliminary results obtained from the implementation of the PAM programme. The objective was to examine the effects of a specifically tailored, group-based, universally delivered, CBT intervention for anxiety in children with visual impairments and to examine the influence of three predictor variables (i.e. age, gender and level of visual impairment) on outcomes.

## Research methods and design

A specifically tailored CBT-based anxiety intervention (PAM programme) for South African children with visual impairments was evaluated in two special schools (School 1 and School 2). The study was set up as a randomised wait-list control group design with pre-, post- and follow-up intervention measures. Participants were randomly assigned to either an immediate intervention group (IIG) or a delayed intervention group (DIG) at their school.

### Participants

All assenting children for whom written parental consent was granted took part. Inclusion criteria required participants to be in grades 4 to 7 (aged between 9 and 14 years), be able to read and write (braille or print) and have no other disability apart from their visual impairment. All children who met inclusion criteria received the PAM programme regardless of their anxiety status (universal prevention).

A final sample of 52 middle-childhood (mean age = 11.46, standard deviation [SD] = 1.4) girls (*n* = 24, 46.15%) and boys (*n* = 28, 53.85%) participated in the study and were followed over a period of 10 months.

### Schools

Participants attended two special schools (School 1 and School 2). These are the only two schools that specifically cater for children who have visual impairments in the Western Cape Province of South Africa – the province where the study was undertaken. The two schools are approximately 110 km apart and differ widely in terms of their geographical location and the availability of resources. The impact of apartheid and its policies have caused severe economic, social and spatial disparities amongst various racial groups in South Africa and this is especially evident at School 1. This school was historically a school for Black children under the Bantu or *Black Education Act* (Act no. 47, 1953) (Union of South Africa 1953), and still today children who attend this school are primarily from disadvantaged communities, with approximately 85% – 90% living below the poverty line, with their parents unemployed and receiving disability grants, and the majority of children receiving government child grants (personal communication, School Psychologist at School 1). The socio-economic circumstances of children at School 2 are similar, as approximately 70% of learners are black or Coloured^1^ and an estimated 55% of children live in poverty and receive government child grants (personal communication, School Psychologist at School 2). The socio-economic situation of these children is in keeping with the general situation in South Africa, where over half of the households in South Africa live below the poverty line (Budlender 2018). However, despite children having similar socio-economic backgrounds, School 2 is situated in a more secure and affluent geographical location. In addition, because School 2 was historically a school for white children (under the *Bantu Education Act*) (Union of South Africa 1953), the school has better infrastructure and facilities. Please note that the racial terms used in this article are controversial in South Africa and have been referred to for the purpose of reporting descriptions between racially different South African communities that exist as a result of the country’s political past. These terms are not used with the intention to be discriminatory.

### Visual impairment

Participants had varying degrees of visual impairment and were categorised as legally blind (*n* = 17) or partially sighted (*n* = 34) (data were missing for one participant). Children categorised as legally blind either had no measurable light perception or had a very limited degree of light perception and experienced difficulties functioning in unfamiliar environments without assistance and could not read printed material.

### Intervention groups

After assenting to participate, the 52 participants were randomly assigned to either an IIG or DIG at their respective school. During data analysis, the IIGs from School 1 and School 2 were combined to form one IIG, and the DIGs from School 1 and School 2 were combined to form one DIG. The combined IIG included 27 participants (15 boys, 12 girls) with a mean age of 11.44 (SD = 1.47); and the combined DIG included 25 participants (13 boys, 12 girls) with a mean age of 11.58 (SD = 1.35).

### Assessments

Participants (*N* = 52) were assessed on four separate occasions. The time line below describes these four assessments for both the IIG and DIG (T1 = Base-line, Time 1):

**Table:** 

Time 1 (T1)	Pre-intervention assessment for the IIG Pre-waitlist assessment for the DIG
Time 2 (T2)	Immediate 1 week post-intervention assessment for the IIG Post-waitlist assessment for the DIG
Time 3 (T3)	3-month follow-up intervention assessment for the IIG Immediate 1-week post-intervention assessment for the DIG
Time 4 (T4)	3-month follow-up assessment for the IIG.

On each occasion two standardised measures were completed:

The *Revised Child Anxiety and Depression 30-Item Scale* (RCADS-30) (Chorpita, Moffitt & Gray 2005; Sandín et al. 2010) is a self-report measure, which assesses anxiety and depression symptoms across six domains: social phobia, separation anxiety, obsessive compulsive disorder, panic disorder, generalised anxiety disorder and major depressive disorder. Participants are asked to rate the frequency with which symptoms occur on a 4-point Likert scale ranging from ‘never’ (scored 0) to ‘always’ (scored 3). A total scale score is obtained by summing the scales’ 30 items yielding a total score of between 0 and 90.

The *Penn State Worry Questionnaire for Children* (PSWQ-C) (Chorpita et al. 1997) is an 11-item questionnaire, which assesses the tendency to engage in excessive generalised and uncontrolled worry. Respondents are asked how often each item applies to them by indicating answer options on a 4-point Likert scale ranging from ‘never true’ (scored 0) to ‘always true’ (scored 3). A total scale score is obtained by summing the scales’ 11 items yielding a score between 0 and 33, with higher scores indicating a greater tendency to worry (Muris, Meesters & Gobel 2001; Stallard et al. 2014).

### Intervention protocol and materials

The PAM programme (Visagie 2016) is a brief CBT-based early intervention and prevention programme for anxiety, specifically tailored to meet the needs of visually impaired children between the ages of 9 and 14 years. Participants received 10 PAM group sessions over the course of 5 weeks. Sessions were delivered twice weekly and lasted approximately 45 min. Sessions were delivered in either English or Afrikaans depending on the children’s language of schooling (English at school 1 and Afrikaans at school 2). During the programme, children participate in activities, which teach coping skills and problem-solving techniques, thereby helping them to deal more effectively with anxiety. Activities teach skills to identify feelings; to learn to relax, to identify unhelpful thoughts and replace them with more helpful thoughts (cognitive restructuring), how to face and overcome daily problems and challenges and how to illicit family and peer support (Barrett, Lowry-Webster & Turner 1999; Stallard et al. 2007; Visagie 2016). The programme uses a tangible soft toy dog (named PAM) with a collar and eight symbolic charms to represent key anxiety management skills. Charms include the following: (1) a heart, a reminder that feelings come from your heart; (2) a hat, a reminder that thoughts come from your head; (3) a butterfly, a reminder of how the body reacts to anxiety (i.e. the butterflies in your tummy); (4) a noodle, a reminder to use relaxation strategies to help the body relax (like a cooked noodle); (5) a musical note, a reminder to do things, which help you to relax and make you feel good (e.g. listen to music, sing a song, play a game, etc.); (6) a shoe, a reminder of the steps to take to face and solve your problems; (7) a star, a reminder to reward yourself for trying your best; and (8) a hand, a reminder to reach out for others when you need help. The format of the programme included large and small group work sessions, role plays, games, stories, activities and quizzes (more information pertaining to the programme’s content can be attained on request).

The 10 sessions of the PAM programme were delivered by the first author with a research facilitator present. The first author is a registered counselling psychologist with ample experience and knowledge relating to developmental psychology and CBT. Research facilitators had at least an honours degree in psychology and had completed a CBT module as part of their academic work. The first author trained and familiarised the facilitators as to the content of the programme. Although they were not required to deliver any sessions individually, they facilitated smaller groups within the larger group with the first author present to provide guidance. Each research facilitator received a copy of the PAM programme manual. The manual describes the goals and strategies for each session, the desired outcomes and the specific activities that are to be completed in each session. To reinforce and generalise sessions, homework tasks were assigned to each session and participants were required to bring completed homework activities to the next session.

### Data collection procedures

Participants were asked to complete three separate measures, the first being a biographical questionnaire, which was only completed at T1, as well as the RCADS-30 and the PSWQ-C (these measures are described in more detail here). The first author and two research facilitators (two postgraduate psychology students) administered the measures in groups at the two identified special schools. Measures were available in English or Afrikaans depending on the children’s language of schooling (English at School 1 and Afrikaans at School 2). Data collection took place in classroom settings at both schools, where participants sat at desks and completed the measures themselves. Data collection was conducted in a manner that accommodated all participants’ specific visual needs (Visagie & Loxton 2014). Questionnaires were read aloud to participants to ensure that questions were correctly understood. Participants were informed that all questionnaire responses were confidential. Participants indicated their answers using their braille machines or enlarged versions of the questionnaires (42.00 cm × 59.40 cm). If a participant required extra attention, one of the researchers assisted him or her individually to complete their questionnaires. Upon completion of the questionnaires, all participants were encouraged to ask any questions that they may have had.

At T2, after all 10 PAM sessions had been delivered to the IIG, the anxiety status of all participants (*N* = 52) (IIG and DIG) was assessed. Data collection procedures occurred similarly to those at T3; after delivering all 10 PAM sessions to the DIG the anxiety status of all participants (*n* = 52) (IIG and DIG) was reassessed. Data collection once again occurred, as with T1 and T2. At T4, 3 months after delivering the PAM programme to the DIG, the anxiety status of all participants (*N* = 52) (IIG and DIG) (that in effect was at 3-month follow-up for the DIG and 6-month follow-up for the IIG) was again assessed.

## Data analysis

The Statistical Package for the Social Sciences (SPSS for Windows version 23.0) (IBM 2015) was used to calculate descriptive and non-parametric statistics. A series of repeated measures of analyses of variance (ANOVA’s) were conducted to compare reports of anxiety within the IIG and DIG across time (T1–T4) and differences between the IIG and DIG at each of the four times of testing were explored using one-way ANOVA. These between-group and within-group effects are reported here.

### Ethical consideration

Permission to conduct the study was obtained from the Western Cape Education Department in South Africa (Reference: 20130507-10635). Ethical approval from Stellenbosch University Research Ethics Committee: Human Research (Humaniora) (HS888/2013) was also obtained. After the two special schools consented to the study, parents were informed of the project via an information sheet and consent form, which they were asked to sign and return. Parental consent was granted for 59 out of a possible 83 children (71%). After enrolment three participants (*n* = 3) withdrew from the study and one participant (*n* = 1) moved to a different school. The remaining 55 participants were followed over a period of 10 months. However, at the time of data analysis, the data of 52 participants could be used. This was because of the necessary exclusion of participants (*n* = 3) from data analysis as a result of one measurement (T1, T2 or T3) being missing.

## Results

### Scores on the Revised Child Anxiety and Depression 30-Item Scale and Penn State Worry Questionnaire for Children

Overall, results indicate that mean base-line scores on the RCADS-30 and PSWQ-C for both groups (IIG and DIG) were slightly higher when compared with post-intervention (T3) scores. At baseline, participants in the IIG (*n* = 27) reported a mean total RCADS-30 score of 27.77 and a mean total PSWQ-C score of 12.84, whilst participants in the DIG (*n* = 25) reported a mean total RCADS-30 score of 34.52 and a mean total PSWQ-C score of 15.32. These initial mean scores were lower than expected and fell below the clinical range on the RCADS-30 of mean scores greater than or equal to 49. At T3, participants in the IIG (*n* = 27) reported a mean total RCADS-30 score of 26.99 and a mean total PSWQ-C score of 11.29, whilst participants in the DIG (*n* = 25) reported a post-intervention (T3) total mean RCADS-30 score of 29.43 and total mean PSWQ-C score of 12.64. Four participants (*n* = 4) displayed elevated anxiety scores at baseline, and at post-intervention (T3) only one participant (*n* = 1) reported anxiety symptoms which fell within the clinical range. This participant was brought to the attention of the school psychologist for further follow-up. Tables 1 and 2 present the means and SD for the IIG (*n* = 27) and DIG (*n* = 25) on the RCADS-30 and PSWQ-C from T1 to T4, respectively.

**TABLE 1a:** Means and standard deviations for the total score on the Revised Child Anxiety and Depression 30-Item Scale for the immediate intervention group (*n* = 27) and delayed intervention group (*n* = 25) from T1 to T4.

Assessment Time (Time)	IIG (*n* = 27)		DIG (*n* = 25)	
	Means	SD	Means	SD
T1	27.77	11.31	34.52	13.76
T2	29.45	13.94	31.75	8.84
T3	26.99	16.64	29.43	11.93

*Source:* Visagie, L.S., 2016, ‘Development, implementation and evaluation of a cognitive behavioural therapy based intervention programme for the management of anxiety symptoms in South African children with visual impairments’, PhD dissertation, Department of Psychology, Stellenbosch University. https://scholar.sun.ac.za/handle/10019.1/100110

Note. T4 = follow-up and there were only 45 participants – IIG (*n* = 23) and DIG (*n* = 22).

IIG, immediate intervention group; DIG, delayed intervention group; SD, standard deviation.

**TABLE 1b:** Means and standard deviations for the total score on the Revised Child Anxiety and Depression 30-Item Scale for the immediate intervention group (*n* = 27) and delayed intervention group (*n* = 25) from T1 to T4.

Assessment Time (Time)	IIG (*n* = 23)		DIG (*n* = 22)	
	Means	SD	Means	SD
T4	27.91	15.16	29.41	12.75

*Source:* Visagie, L.S., 2016, ‘Development, implementation and evaluation of a cognitive behavioural therapy based intervention programme for the management of anxiety symptoms in South African children with visual impairments’, PhD dissertation, Department of Psychology, Stellenbosch University. https://scholar.sun.ac.za/handle/10019.1/100110

Note. T4 = follow-up and there were only 45 participants – IIG (*n* = 23) and DIG (*n* = 22).

IIG, immediate intervention group; DIG, delayed intervention group; SD, standard deviation.

**TABLE 2a:** Means and standard deviations for the total score on the Penn State Worry Questionnaire for Children for the immediate intervention group (*n* = 27) and delayed intervention group (*n* = 25) from T1 to T4.

Assessment Time (Time)	IIG (*n* = 27)		DIG (*n* = 25)	
	Means	SD	Means	SD
T1	12.84	5.95	15.32	5.91
T2	11.31	5.97	14.33	6.31
T3	11.29	6.44	12.64	4.79

*Source:* Visagie, L.S., 2016, ‘Development, implementation and evaluation of a cognitive behavioural therapy based intervention programme for the management of anxiety symptoms in South African children with visual impairments’, PhD dissertation, Department of Psychology, Stellenbosch University. https://scholar.sun.ac.za/handle/10019.1/100110

Note. T4 = follow-up and there were only 45 participants – IIG (*n* = 23) and DIG (*n* = 22).

IIG, immediate intervention group; DIG, delayed intervention group; SD, standard deviation.

**TABLE 2b:** Means and standard deviations for the total score on the Penn State Worry Questionnaire for Children for the immediate intervention group (*n* = 27) and delayed intervention group (*n* = 25) from T1 to T4.

Assessment Time (Time)	IIG (*n* = 23)		DIG (*n* = 22)	
	Means	SD	Means	SD
T4	11.04	7.02	12.41	7.25

*Source:* Visagie, L.S., 2016, ‘Development, implementation and evaluation of a cognitive behavioural therapy based intervention programme for the management of anxiety symptoms in South African children with visual impairments’, PhD dissertation, Department of Psychology, Stellenbosch University. https://scholar.sun.ac.za/handle/10019.1/100110

Note. T4 = follow-up and there were only 45 participants – IIG (*n* = 23) and DIG (*n* = 22).

IIG, immediate intervention group; DIG, delayed intervention group; SD, standard deviation.

Overall 2 (groups) × 4 (time points) ANOVAs were performed on the total scores of the RCADS-30 and PSWQ-C for the IIG (*n* = 23) and DIG (*n* = 22) separately. The multivariate main effects for time on both these measures were non-significant for both the IIG and DIG on either the RCADS-30: (*F*[3, 20] = 0.481, *p* = 0.699 [IIG]: *F*[3, 19] = 1.202, *p* = 0.336 [DIG]) or PSWQ-C: (*F*[3, 20] = 1.538, *p* = 0.235 [IIG]: *F*(3, 19) = 1.408, *p* = 0.271 [DIG]).

### Within group and between effects

Results of the multivariate main effects for time were not significant for both the IIG and DIG on either the RCADS-30 (*F*[3, 20] = 0.481, *p* = 0.699 [IIG]: F(3, 19) = 1.202, *p* = 0.336 [DIG]) or the PSWQ-C (F[3, 20] = 1.538, *p* = 0.235 [IIG]: *F*(3, 19) = 1.408, *p* = 0.271 [DIG]). There were no significant between group effects.

### Total group effects

When taking the statistical sample as a whole (*N* = 52) into account, there was a significant decline in the total worry score on the PSWQ-C from pre- (T1) to post- (T3) test (*F*[1, 51] = 4.436, *p* = 0.040). Results on the RCADS-30 for the total sample (*N* = 52) from T1 to T3 were non-significant (*F*[1, 51] = 2.347, *p* = 0.132). Effects on the six sub-scales of the RCADS-30 from T1 to T3 were also non-significant (*F*[6, 46] = 1.080, *p* = 0.388).

### Effects of predictor variables

When considering the variables of age (younger 9–11-year-old vs. older 12–14-year-old children), gender (girls vs. boys) and level of visual impairment (legally blind vs. partially sighted), the following was noted:

In terms of age, there was a significant interaction between time and age for younger children (*n* = 30) on the RCADS-30 (*F*[1, 29] = 11.771, *p* = 0.002). Thus, the younger participants’ mean score at T3 (*M* = 24.85) was significantly lower than at T1 (*M* = 32.68). Multivariate repeated measures ANOVA’s were also performed on the six subscale scores of the RCADS-30. There was a significant multivariate main effect for the younger participants (*F*[6, 24] = 2.976, *p* = 0.026). Post hoc comparisons with Bonferroni adjustments indicated significant reductions on the major depression (*p* = 0.001) and obsessive compulsive disorder (*p* = 0.003) subscales. Results on the PSWQ-C relating to age were non-significant.

Anxiety scores on the RCADS-30 and worry scores on the PSWQ-C for boys (*n* = 28) and girls (*n* = 24) were also compared. Results on the RCADS-30 were non-significant for both boys and girls). However, results on the PSWQ-C were significant for girls with (*F*[1, 23] = 13.411, *p* = 0.001) with the mean score at T3 (*M* = 11.67) being significantly lower than at T1 (*M* = 15.77).

Total mean scores on the RCADS-30 and PSWQ-C for the legally blind (*n* = 17) and partially sighted (*n* = 34) groups were also compared. There was a significant interaction between time and vision for legally blind participants on the RCADS-30 (*F*[1, 16] = 7.845, *p* = 0.013). Thus, for the legally blind group the mean score at T3 (*M* = 24.22) was significantly lower than at T1 (*M* = 31.41). Multivariate repeated measures ANOVA’s were also performed on the six subscale scores of the RCADS-30 with a significant effect for the legally blind group (*F*[6, 11] = 4.55, *p* = 0.048). Post hoc comparisons with Bonferroni adjustments indicated that for the legally blind group the mean score on major depression at T3 (*M* = 3.24) was significantly (*p* = 0.016) lower than at T1 (*M* = 4.74), and the mean score on obsessive compulsive disorder at T3 (*M* = 3.88) was significantly (*p* = 0.014) lower than at T1 (*M* = 6.79). Results on the PSWQ-C were non-significant.

## Discussion

The purpose of this study was to explore the effect of a specifically tailored CBT-based programme (PAM programme) on participant-reported symptoms of anxiety.

The PAM programme did not lead to post intervention reductions in self-reported anxiety and worry scores on the RCADS-30 and PSWQ-C. These non-significant results may relate to the fact that base-line anxiety symptoms were unexpectedly low. The overall mean score within each group virtually remained unchanged from T1 to T4.

A second aim of the present study was to examine the effects of the predictor variables of age, gender and level of visual impairment. Younger (9–11-year-olds) and legally blind participants reported significant reductions in anxiety scores on the RCADS-30 from pre- (T1) to post- (T3) test. Girls also reported a significant reduction in worry scores on the PSWQ-C. Thus, although there were no overall effects, it appears that the PAM programme may be beneficial for girls, younger participants (aged 9–11) and legally blind children. Results relating to the legally blind group are particularly noteworthy, as children with severe visual impairments have been identified as a high-risk group (Loxton, Visagie & Ollendick 2012; Visagie et al. 2013, 2015). It is also encouraging to note the effects relating to a decrease in symptoms of major depression for younger (age 9–11-year-olds) and legally blind children. These results are important as it has been noted that some larger scale depression prevention studies have recently found non-significant universal effects (Herman et al. 2009; Rowling & Kasunic 2006; Stewart 2008; Taylor et al. 2014).

The absence of an overall effect could be masked by two factors. Firstly, the sample size is small resulting in reduced statistical power to detect small differences. Secondly, initial levels of anxiety were unexpectedly low and, with the exception of four participants, all were in the ‘normal’ range at base-line. Thus, the groups could not be expected to differ substantially after implementation of the PAM programme. This is a limitation of universal approaches and raises the question of whether it is worth the time and money to offer a programme to a whole group of children if the majority are not anxious (Rose, Miller & Martinez 2009). According to a public health universal approach, even small effects can be meaningful, as decreasing the distribution of symptoms in the population by even a small amount may often correspond to a reduction in the occurrence of overall cases of disorder (Andrews, Szabo & Burns 2002; Mychailyszyn et al. 2012).

Another limitation relates to the lack of multiple-informants for data collection. Previous studies have noted non-significant results post-intervention on participant self-reports, however, when considering other anxiety measures (e.g. diagnostic interviews and parent or teacher reports) significant intervention effects were noted (Bernstein et al. 2005, 2008; Ginsburg 2009; Nauta et al. 2003; Urao et al. 2016; Wood et al. 2009). This may have also been the case in this study, but because additional teacher reports to assess intervention outcomes were not returned, these sources of data could not be included.

A further limitation relates to the absence of a control group to assess for possible changes being the result of the natural passing of time (i.e. maturation). This would have been ideal, but as a result of the target population already being so limited in size, the inclusion of a control group was not feasible. This would have decreased numbers of participants in the IIG and DIG even further.

Lastly, it may also be that significant effects may present with ensuing time as children become more adept at using their newly learnt anxiety management skills (Mostert & Loxton 2008).

## Conclusion

Our results indicate that the PAM programme did not bring about a significant decrease in symptoms of anxiety within a group of visually impaired children. However, our results suggest that the PAM programme may be more beneficial for girls, younger participants (aged 9–11) and legally blind children.

The present study is the first of its kind to evaluate the effects of a specifically tailored CBT-based anxiety intervention programme for children with visual impairments. This population has been grossly neglected in previous child anxiety research, and despite limited results our study provides an insight into how traditional CBT interventions can be adapted for use with this group. Participants perceived the PAM programme to be enjoyable and helpful, as was evident from their enthusiasm and impromptu responses throughout programme sessions. Results obtained from this study provide a good foundation upon which future up-dated anxiety intervention programmes can be built. Thus, continued research in the area of anxiety intervention and prevention for this population should be promoted.

## Footnotes

1 We use terminology in current South African equity legislation. We do not believe that these categories have any genetic or scientific basis, but the categories have social meaning in contemporary South Africa.

## References

[CIT0001] Ahlen, J., Breitholtz, E., Barrett, P.M. & Gallegos, J, 2012, ‘School-based prevention of anxiety and depression: A pilot study in Sweden’, *Advances in School Mental Health Promotion* 5(4), 246–257. 10.1080/1754730X.2012.730352

[CIT0002] Andrews, G., Szabo, M. & Burns, J, 2002, ‘Preventing major depression in young people’, *The British Journal of Psychiatry* 181(6), 460–462. 10.1192/bjp.181.6.460 12456512

[CIT0003] Barrett , P.M, 2005, *Friends for life*, Australian Academic Press, Brisbane.

[CIT0004] Barrett, P.M., Lowry-Webster, H. & Turner, C.M, 1999, *FRIENDS for children participant workbook*, Australian Academic Press, Brisbane.

[CIT0005] Bernstein, G.A., Bernat, D.H., Victor, A.M. & Layne, A.E, 2008, ‘School-based interventions for anxious children: 3-, 6-, and 12-month follow-ups’, *Journal of the American Academy of Child & Adolescent Psychiatry* 47(9), 1039–1047. 10.1097/CHI.0b013e31817eecc0 18665000 PMC3427760

[CIT0006] Bernstein, G.A., Layne, A.E., Egan, E.A. & Tennison, D.M, 2005, ‘School-based interventions for anxious children’, *Journal of the American Academy of Child & Adolescent Psychiatry* 44(11), 1118–1127. 10.1097/01.chi.0000177323.40005.a1 16239860 PMC2442034

[CIT0007] Bittner, A., Egger, H.L., Erkanli, A., Jane Costello, E., Foley, D.L. & Angold, A, 2007, ‘What do childhood anxiety disorders predict?’, *Journal of Child Psychology and Psychiatry* 48(12), 1174–1183. 10.1111/j.1469-7610.2007.01812.x 18093022

[CIT0008] Budlender, D, 2018, ‘Income support for children in the context of poverty and inequality’, in K. Hall, L. Richter, Z. Mokomane & L. Lake (eds.), *South African child gauge 2018*, pp. 93–100, Children’s Institute, University of Cape Town, Cape Town.

[CIT0009] Campbell, M.A, 2003, ‘Prevention and intervention for anxiety disorders in children and adolescents: A whole school approach’, *Australian Journal of Guidance and Counselling* 13(1), 47–62. 10.1017/S1037291100004738

[CIT0010] Chorpita, B.F., Moffitt, C.E. & Gray, J, 2005, ‘Psychometric properties of the revised child anxiety and depression scale in a clinical sample’, *Behaviour Research and Therapy* 43(3), 309–322. 10.1016/j.brat.2004.02.004 15680928

[CIT0011] Chorpita, B.F., Tracey, S.A., Brown, T.A., Collica, T.J. & Barlow, D.H, 1997, ‘Assessment of worry in children and adolescents: An adaptation of the Penn State Worry Questionnaire’, *Behaviour Research and Therapy* 35(6), 569–581. 10.1016/S0005-7967(96)00116-7 9159982

[CIT0012] Donovan, C.L. & Spence, S.H, 2000, ‘Prevention of childhood anxiety disorders’, *Clinical Psychology Review* 20(4), 509–531. 10.1016/S0272-7358(99)00040-9 10832552

[CIT0013] Ginsburg, G.S, 2009, ‘The child anxiety prevention study: Intervention model and primary outcomes’, *Journal of Consulting and Clinical Psychology* 77(3), 580–587. 10.1037/a0014486 19485597 PMC3373966

[CIT0014] Gullone, E, 1996, ‘Normal fear in people with a physical or intellectual disability’, *Clinical Psychology Review* 16(8), 689–706. 10.1016/S0272-7358(96)00041-4

[CIT0015] Herman, K.C., Reinke, W., Parkin, J., Traylor, K. & Agarwal, G, 2009, ‘Childhood depression: Rethinking the role of the school’, *Psychology in the Schools* 46(5), 433–446. 10.1002/pits.20388

[CIT0016] IBM, 2015, *SPSS statistics*, 230th edn., IBM Corp, Armonk, NY.

[CIT0017] Johnston, K.M., Kemps, E. & Chen, J, 2018, ‘A meta-analysis of universal school-based prevention programs for anxiety and depression in children’, *Clinical Child and Family Psychology Review* 21(4), 466–481. 10.1007/s10567-018-0266-5 30105480

[CIT0018] Kendall, P.C, 1990, *The coping cat workbook*, Temple University, Merion Station, PA.

[CIT0019] Korkodilos, M, 2016, *The mental health of children and young people in England*, Public Health England, London.

[CIT0020] Last, C.G., Perrin, S., Hersen, M. & Kazdin, A.E, 1996, ‘A prospective study of childhood anxiety disorders’, *Journal of the American Academy of Child & Adolescent Psychiatry* 35(11), 1502–1510. 10.1097/00004583-199611000-00019 8936917

[CIT0021] Lawrence, D., Johnson, S., Hafekost, J., Boterhoven de Haan, K., Sawyer, M., Ainley, J. et al., 2015, *The mental health of children and adolescents*, Department of Health, Canberra.

[CIT0022] Liddle, I. & Macmillan, S, 2010, ‘Evaluating the FRIENDS programme in a Scottish setting’, *Educational Psychology in Practice* 26(1), 53–67. 10.1080/02667360903522785

[CIT0023] Loxton, H., Visagie, L. & Ollendick, T.H, 2012, ‘Assessing the fears of children with varying degrees of visual impairment’, in 30th International Congress of Psychology, Cape Town, South Africa, July 23–27, 2012.

[CIT0024] McLoone, J., Hudson, J.L. & Rapee, R.M, 2006, ‘Treating anxiety disorders in a school setting’, *Education and Treatment of Children* 29(2), 219–242. https://www.jstor.org/stable/42899883

[CIT0025] Merikangas, K.R., He, J.P., Burstein, M., Swanson, S.A., Avenevoli, S., Cui, L. et al., 2010, ‘Lifetime prevalence of mental disorders in US adolescents: Results from the National Comorbidity Study-Adolescent Supplement (NCS-A)’, *Journal of the American Academy of Child and Adolescent Psychiatry* 49(10), 980–989. 10.1016/j.jaac.2010.05.017 20855043 PMC2946114

[CIT0026] Mostert, J. & Loxton, H, 2008, ‘Exploring the effectiveness of the FRIENDS program in reducing anxiety symptoms among South African children’, *Behaviour Change* 25(2), 85–96. 10.1375/bech.25.2.85

[CIT0027] Mrazek, P.J. & Haggerty, R.J, 1994, *Reducing risks for mental disorders: Frontiers for preventive intervention research*, National Academies Press, Washington, DC. 25144015

[CIT0028] Muris, P., Meesters, C. & Gobel, M, 2001, ‘Reliability, validity, and normative data of the Penn State Worry Questionnaire in 8-12-yr-old children’, *Journal of Behavior Therapy and Experimental Psychiatry* 32(2), 63–72. 10.1016/S0005-7916(01)00022-2 11764062

[CIT0029] Mychailyszyn, M.P., Brodman, D.M., Read, K.L. & Kendall, P.C, 2012, ‘Cognitive-behavioural school-based interventions for anxious and depressed youth: A meta-analysis of outcomes’, *Clinical Psychology Science and Practice* 19(2), 129–153. 10.1111/j.1468-2850.2012.01279.x

[CIT0030] Nauta, M.H., Scholing, A., Emmelkamp, P.M. & Minderaa, R.B, 2003, ‘Cognitive-behavioral therapy for children with anxiety disorders in a clinical setting: No additional effect of a cognitive parent training’, *Journal of the American Academy of Child and Adolescent Psychiatry* 42(11), 1270–1278. 10.1002/cpp.314 14566163

[CIT0031] Ollendick, T.H., Matson, J.L. & Helsel, W.J, 1985, ‘Fears in visually-impaired and normally-sighted youths’, *Behaviour Research and Therapy* 23(3), 375–378. 10.1016/0005-7967(85)90017-8 4004716

[CIT0032] Pina, A.A., Silverman, W.K., Weems, C.F., Kurtines, W.M. & Goldman, M.L, 2003, ‘A comparison of completers and noncompleters of exposure-based cognitive and behavioral treatment for phobic and anxiety disorders in youth’, *Journal of Consulting and Clinical Psychology* 71(4), 701–705. 10.1037/0022-006X.71.4.701 12924675

[CIT0033] Prinzie, P., Van Harten, L.V., Dekovic, M., Van den Akker, A.L. & Shiner, R.L, 2014, ‘Developmental trajectories of anxious and depressive problems during the transition from childhood to adolescence: Personality x parenting interactions’, *Development and Psychopathology* 26(11), 1077–1092. 10.1017/S0954579414000510 24914625

[CIT0034] Rey, Y., Marin, C.E. & Silverman, W.K, 2011, ‘Failures in cognitive-behavior therapy for children’, *Journal of Clinical Psychology* 67(11), 1140–1150. 10.1002/jclp.20848 21953495

[CIT0035] Rose, H., Miller, L. & Martinez, Y, 2009, ‘“FRIENDS for life”: The results of a resilience-building, anxiety-prevention program in a Canadian elementary school’, *Professional School Counselling* 12(6), 400–407. 10.5330/PSC.n.2010-12.400

[CIT0036] Rowling, L. & Kasunic, V, 2006, ‘Prevention of depression in young people: An Australian perspective’, *Clinical Neuropsychiatry* 3(1), 29–38.

[CIT0037] Sandín, B., Chorot, P., Valiente, R.M. & Chorpita, B.F, 2010, ‘Development of a 30-item version of the Revised Child Anxiety and Depression Scale’, *Revista De Psicopatología y Psicología Clínica* 15(3), 165–178. 10.5944/rppc.vol.15.num.3.2010.4095

[CIT0038] Silverman, W.K., Pina, A.A. & Viswesvaran, C, 2008, ‘Evidence-based psychosocial treatments for phobic and anxiety disorders in children and adolescents’, *Journal of Clinical Child and Adolescent Psychology* 37(1), 105–130. 10.1080/15374410701817907 18444055

[CIT0039] Stallard, P, 2010, ‘Mental health prevention in UK classrooms: The FRIENDS anxiety prevention programme’, *Emotional and Behavioural Difficulties* 15(1), 23–35. 10.1080/13632750903512381

[CIT0040] Stallard, P., Simpson, N., Anderson, S., Hibbert, S. & Osborn, C, 2007, ‘The FRIENDS emotional health programme: Initial findings from a school-based project’, *Child and Adolescent Mental Health* 12(1), 32–37. 10.1111/j.1475-3588.2006.00421.x 32811032

[CIT0041] Stallard, P., Skryabina, E., Taylor, G., Phillips, R., Daniels, H., Anderson, R. et al., 2014, ‘Classroom-based cognitive behaviour therapy (FRIENDS): A cluster randomised controlled trial to prevent anxiety in children through education in schools (PACES)’, *The Lancet Psychiatry* 1(3), 185–192. 10.1016/S2215-0366(14)70244-5 26360730

[CIT0042] Stewart, D, 2008, ‘Implementing mental health promotion in schools: A process evaluation’, *The International Journal of Mental Health Promotion* 10(1), 32–41. 10.1080/14623730.2008.9721755

[CIT0043] Taylor, J.A., Phillips, R., Cook, E., Georgiou, L., Stallard, P. & Sayal, K, 2014, ‘A qualitative process evaluation of classroom-based cognitive behaviour therapy to reduce adolescent depression’, *International Journal of Environmental Research and Public Health* 11(6), 5951–5969. 10.3390/ijerph110605951 24905241 PMC4078557

[CIT0044] Union of South Africa, 1953, *Black Education Act (Act no. 47, 1953)*, Government Printer, Pretoria, viewed 26 February 2019, from https://www.sahistory.org.za/archive/bantu-education-act-act-no-47-1953.

[CIT0045] Urao, Y., Yoshinaga, N., Asano, K., Ishikawa, R., Tano, A., Sato, Y. et al., 2016, ‘Effectiveness of a cognitive behavioural therapy-based anxiety prevention programme for children: A preliminary quasi-experimental study in Japan’, *Child and Adolescent Psychiatry and Mental Health* 10(1), 1 10.1186/s13034-016-0091 26884810 PMC4754865

[CIT0046] Visagie, L. & Loxton, H, 2014, ‘Child-friendly procedures and accommodations for the use of a self-report fear survey with children who have visual impairments: Reflections on a South African case study’, *The British Journal of Visual Impairment* 32(3), 179–190. 10.1177/0264619614535373

[CIT0047] Visagie, L., Loxton, H., Ollendick, T.H. & Steel, H, 2013, ‘Comparing fears in South African children with and without visual impairments’, *Journal of Visual Impairment & Blindness* 107(3), 193–205. 10.1177/0145482X1310700304

[CIT0048] Visagie, L., Loxton, H. & Silverman, W.K, 2015, ‘Research protocol: Development, implementation and evaluation of a cognitive behavioural therapy-based intervention programme for the management of anxiety symptoms in South African children with visual impairments’, *African Journal of Disability* 4(1), 1–10. 10.4102/ajod.v4i1.160 PMC5433474 28730026

[CIT0049] Visagie, L., Loxton, H., Stallard, P. & Silverman, W.K, 2017, ‘Insights into feelings, thoughts and behaviours from children with visual impairments: A focus group study prior to adapting a CBT-based anxiety intervention’, *Journal of Visual Impairment & Blindness* 111(3), 231–246. 10.1177/0145482X1711100304

[CIT0050] Visagie, L.S, 2016, ‘Development, implementation and evaluation of a cognitive behavioural therapy based intervention programme for the management of anxiety symptoms in South African children with visual impairments’, PhD dissertation, Department of Psychology, Stellenbosch University, Stellenbosch https://scholar.sun.ac.za/handle/10019.1/100110 10.4102/ajod.v4i1.160 PMC5433474 28730026

[CIT0051] Walkup, J.T., Albano, A.M., Piacentini, J., Birmaher, B., Compton, S.N., Sherrill, J.T. et al., 2008, ‘Cognitive behavioral therapy, sertraline, or a combination in childhood anxiety’, *The New England Journal of Medicine* 359(26), 2753–2766. 10.1097/01.chi.0000162583.25829.4b 18974308 PMC2702984

[CIT0052] Weems, C.F. & Silverman, W.K, 2013, ‘Anxiety disorders’, in T.P. Beauchaine & T.P. Hinshaw (eds.), *Child and adolescent psychopathology*, 2nd edn., pp. 513–542, John Wiley & Sons, New York, NY.

[CIT0053] Weimer, S.A. & Kratochwill, T.R, 1991, ‘Fears of visually impaired children’, *Journal of Visual Impairment & Blindness* 85(3), 118–124. 10.1177/0145482X9108500308

[CIT0054] Wergeland, G.J.H., Fjermestad, K.W., Marin, C.E., Haugland, B.S.-M., Silverman, W.K., Öst, L.-G. et al., 2015, ‘Predictors of dropout from community clinic child CBT for anxiety disorders’, *Journal of Anxiety Disorders* 31, 1–10. 10.1016/j.janxdis.2015.01.004 25637909

[CIT0055] Wood, J.J., McLeod, B.D., Piacentini, J.C. & Sigman, M, 2009, ‘One-year follow-up of family versus child CBT for anxiety disorders: Exploring the roles of child age and parental intrusiveness’, *Child Psychiatry and Human Development* 40(2), 301–316. 10.1007/s10578-009-0127-z 19165592 PMC2779419

[CIT0056] World Health Organization, 2004, *Prevention of mental disorders: Effective interventions and policy options*, World Health Organization, Geneva, viewed 28 August 2020, from https://www.who.int/mental_health/evidence/en/prevention_of_mental_disorders_sr.pdf.

